# Stiffness and Elasticity of Aorta Assessed Using Computed Tomography Angiography as a Marker of Cardiovascular Health—A Cross-Sectional Study

**DOI:** 10.3390/jcm13020384

**Published:** 2024-01-10

**Authors:** Wojciech Hajdusianek, Aleksandra Żórawik, Piotr Macek, Małgorzata Poręba, Rafał Poręba, Paweł Gać

**Affiliations:** 1 Department of Population Health, Division of Environmental Health and Occupational Medicine, Wroclaw Medical University, Mikulicza-Radeckiego 7, 50-368 Wroclaw, Poland; 2 Department of Internal Medicine, Occupational Diseases, Hypertension and Clinical Oncology, Wroclaw Medical University, Borowska 213, 50-556 Wroclaw, Poland; 3 Department of Paralympic Sports, Wroclaw University of Health and Sport Sciences, Witelona 25a, 51-617 Wroclaw, Poland; 4 Centre of Diagnostic Imaging, 4th Military Hospital, Weigla 5, 50-981 Wroclaw, Poland

**Keywords:** aortic elasticity, aortic stiffness, cardiovascular health, computed tomography angiography

## Abstract

Cardiovascular (CV) health can be measured using the American Health Association’s Life’s Simple 7 scale (ALS7). Aortic stiffness (AoS) and elasticity (AoE) can be assessed using various methods, e.g., computed tomography (CT). To measure AoE, we use aortic strain and distensibility (AoD). The aim of this study was to examine the relationship between ALS7, AoS, and AoE. The study group (SG) was composed of 96 patients (mean age 70.41 ± 8.32 years) with a BMI of 25.58 ± 3.12 kg/m^2^; 28.1% were smokers, 54.2% had hypertension, 11.4% had diabetes, and 67.7% had hypercholesterolemia. The SG was further divided into three subgroups (optimal (ALS7-H), intermediate (ALS7-I), and inadequate (ALS7-L)) based on the ALS7. The AoS and AoE were assessed in each of them. We found that the ALS7-I and ALS7-H had significantly lower AoS values compared to the ALS7-L (AoS: 3.50 ± 0.53 and 4.10 ± 0.70 vs. 4.57 ± 1.03, respectively). The opposite relationship was observed for AoE measured with AoD in the ALS7-H vs. ALS7-L (AoD: 0.23 ± 0.14 vs. 0.11 ± 0.09 cm^2^/dyn). AoS correlated (r = 0.61) with systolic blood pressure (BP). In our regression model, higher scores on the ALS7 in BP, smoking, and BMI were independent protective factors against greater AoS. Higher ALS7 scores in BP, smoking, BMI, and physical activity were protective factors against lesser aortic strain. Higher scores in ALS7 for BP and smoking were protective factors against lesser AoD. We conclude that better cardiovascular health expressed via higher scores obtained on the ALS7 is associated with lower AoS and higher AoE on CT.

## 1. Introduction

Cardiovascular diseases (CVDs) are a leading cause of death, reported by many scientists from different countries. They were found to be the cause of 31.5% of all deaths and 45% of all non-communicable disease deaths. Due to CVDs, approximately 4 million people die in Europe each year, and, among them, 2.2 million are women. Amongst CVDs, the most prevalent diseases are coronary heart disease and stroke. The mean length of stay after myocardial infarction is 6.7 days amongst European OECD countries [1]. Similarly, they were found to be a major cause of death in the United States [2] and the Chinese population [3]. Therefore, cardiovascular health is very important to public health. We can define cardiovascular health not only as a lack of disease but also as an estimation of the probability of the occurrence of CVDs in the future, which we can assess by evaluating particular risk factors [4,5,6,7,8,9,10]. In our research, to study cardiovascular health, we decided to use the Life’s Simple 7 scale, which was designed by the American Heart Association [11,12,13].

In our research, we sought to study aortic stiffness and elasticity as a possible new marker of cardiovascular health. Descriptions of aortic stiffness and elasticity measurement methods and their importance were discussed in our previous article [14], and the most significant findings in the context of this study were as follows: increased aortic stiffness is thought to be a predictor of coronary artery disease [15] and it has been associated with increased blood pressure [16] or arterial calcification [17,18,19,20], whereas impaired aortic elasticity has been associated with prehypertension [21].

The aim was to study the relationship between stiffness and the elasticity of the aorta measured using the aortic stiffness index and aortic strain and distensibility, which were measured in coronary computed tomography angiography (CCTA) using cardiovascular health, measured using the American Heart Association Life’s Simple 7 classification scale in a group of patients who already had medical indications to prompt radiological examination. The expected primary outcome was that aortic stiffness and aortic elasticity were associated with cardiovascular health. The expected secondary outcomes were the determination of the particular Life 7 factors associated with AoS and AoE.

## 2. Materials and Methods

This study was part of the project “Cardiovascular health and risk markers assessed by diagnostic imaging in patients with hypertension and obstructive sleep apnea”. To ensure an appropriate level of statistical power, a sample size calculator was used to calculate the required number of participants to be included in the study. The following conditions were used for calculations: confidence level 95%, sample proportion 50%, maximal error 10%, and population size 2.8 million. The sample size was estimated at 96 participants.

The inclusion criteria were as follows. All participants must have had a medical indication to conduct a CCTA, been at least 18 years old (which is the age of the majority in Poland), and provided informed consent to participate in the study. In total, 106 patients met the inclusion criteria. Subsequently, the exclusion criteria were insufficient quality of CCTA and being previously diagnosed with coronary artery disease, stroke, chronic kidney disease, and hypothyroidism. Due to the exclusion of 9 participants from the study, the population was composed of 96 participants.

We used the AHA’s Life’s Simple 7 classification (ALS7) to study cardiovascular health. It is a scale composed of 7 modifiable risk factors, which are smoking, body mass index, physical activity, a healthy diet, total cholesterol, blood pressure, and the fasting plasma glucose concentration. Each of these factors is assessed, and the result of this evaluation reflects one of three grades: inadequate (sometimes described as “poor”), average (sometimes described as “intermediate”), and optimal (sometimes described as “ideal”). In scientific analysis, the ALS7 can be assessed in the following manner: each of the cardiovascular health factors is assigned a value 0, 1, or 2 when meeting the criteria of the inadequate, average, and optimal grades, respectively, and the values are summed. The interpretation of the values’ total sum is slightly different among various researchers, e.g., Folsom et al. [11] and Hasbani et al. [12] considered 0–4 as inadequate, 5–9 as average, and 10–14 as optimal, whereas Desai et al. considered values < 8 as poor, 9–11 as intermediate, and >12 as ideal [13].

The measurements of aorta were conducted in the following manner. The aortic diameter was measured using the multiplanar reconstruction of computed tomography in a cross-section approximately 3.0 cm above the ring of the valve of the aorta and perpendicular to the long axis of the vessel. The phase of the heart cycle was monitored using electrocardiography. Accordingly, the aortic systolic diameter was assessed during the full opening of the aortic valve. Subsequently, the diastolic diameter of the aorta was measured at the top of the R-wave present in the recorded electrocardiography.

Aortic stiffness and elasticity measurements were performed similarly to our previous research [22] and were based on the following Equations (1)–(3):

(1) $$Ao\;stiffness\;index=\frac{\ln\left(\frac{systolic\;blood\;pressure}{diastolic\;blood\;pressure}\right)}{\frac{Ao\;systolic\;diameter-Ao\;diastolic\;diameter}{Ao\;diastolic\;diameter}}$$

(2) $$Ao\;strain=\left(\frac{Ao\;systolic\;diameter-Ao\;diastolic\;diameter}{Ao\;diastolic\;diameter}\right)\times 100\;$$

(3) $$Ao\;distensibility=\frac{2\times Ao\;strain}{systolic\;blood\;pressure-diastolic\;blood\;pressure}\;$$

Equation (1) was used to calculate the aortic (Ao) stiffness index, which was used to assess aortic stiffness. As can be seen from the equation, it depends on the systolic and diastolic blood pressure in relation to the aortic systolic and diastolic diameter. The aortic elasticity assessment was based on aortic strain and distensibility. Aortic distensibility (Equation (3)) is a result of the division of the aortic strain by the difference between the systolic and diastolic blood pressure. Aortic strain (Equation (2)) represents the relationship between the change in aortic diameter in the systole and diastole relative to the aortic diastolic diameter.

Statistical analysis was performed with the Statistica 13 (version 13.1.336.0) TIBCO Software Inc. (Palo Alto, CA, USA) provided by StatSoft Poland. Quantitative analysis consisted of the analysis of the distribution and verification of hypotheses with appropriately selected statistical tests. When comparing subgroups, a normal distribution was assessed in each one of them. The distribution of variables was firstly assessed visually by plotting histograms with a normal distribution curve; then, distribution parameters such as the mean, median, skewness, and kurtosis were assessed. The Shapiro–Wilk statistical test of normality was applied, with the level of statistical significance taken as *p* = 0.05. Subsequently, after normality assessment, further statistical tests for group comparison were applied. Firstly, the assumptions of the tests were checked. For data that were not normally distributed, a Mann–Whitney U test was performed. When, in a given analysis, more than two groups were assessed, i.e., more than one statistical test was conducted, the Bonferroni correction method was applied to counteract the problem of multiple comparisons. In further analyses, we built three multiple regression models. Each time, the regression assumptions were verified. We used stepwise backward regression analysis to build multivariable models. All results were considered relevant at a significance level of *p* < 0.05.

## 3. Results

The studied group was composed of 96 patients: 52 men (54.2%) and 44 women (45.8%), with a mean age 70.41 ± 8.32 years. The mean height of participants was 166.90 ± 7.80 cm, the mean body mass was 71.52 ± 11.68 kg, and the mean BMI was 25.58 ± 3.12 kg/m^2^. Among the participants, 28.1% declared that they were smoking and 67.7% were diagnosed with hypercholesterolemia. The mean value of total cholesterol in the studied group was 224.61 ± 43.25 mg/dL. Due to 11.4% of participants being diagnosed with diabetes type 2, the mean measured fasting glucose was 120.27 ± 49.31 mg/dL. More than half (54.2%) of the participants were diagnosed with arterial hypertension. Accordingly, the mean systolic and diastolic blood pressure were 140.05 ± 18.56 and 86.04 ± 9.15 mmHg, respectively. The most common indications for CCTA were suspected chronic coronary artery disease (58.3%), chest pain (51%), and coronary artery disease risk factors. The characteristics of the studied group are shown in Table 1.

Among the 96 participants, the worst behavioral pattern was seen in 27 (28.1%) participants who were smoking at the time, 11 (11.4%) who had a BMI of more than 30 kg/m^2^, 34 (35.4%) who did not perform any physical activity, and 37 (38.5%) who had poor diet scores. The laboratory-assessed cardiovascular risk factors were total cholesterol, blood pressure, and fasting glucose. The worst results were as follows: 29 (30.2%) patients had at least 240 mg/dL of total cholesterol, 59 (61.4%) participants had systolic blood pressure of at least 140 mmHg and/or diastolic blood pressure of at least 90 mmHg, and 21 (21.9%) participants had fasting glucose of 126 mg/dL or more. The results of the AHA’s Life’s Simple 7 classification among all participants are presented in Table 2.

The obtained mean ALS7 cardiovascular health score (CVH score) was 6.56 ± 1.98 points. Among the participants, 5 (5.2%) had optimal CVH scores, 70 (72.9%) had average scores, and 21 (21.9%) presented inadequate scores. Table 3 presents the coronary computed tomography angiography parameters of our studied group. The mean coronary artery calcium score was 194.18 ± 59.48, and the mean aortic systolic and diastolic diameters were 34.39 ± 4.39 and 33.33 ± 4.26 mm, respectively, whereas the mean aortic stiffness index, strain, and distensibility were 4.17 ± 0.80, 3.20 ± 2.08 (%), and 0.14 ± 0.13 (cm^2^/dyn).

**Figure 1:**
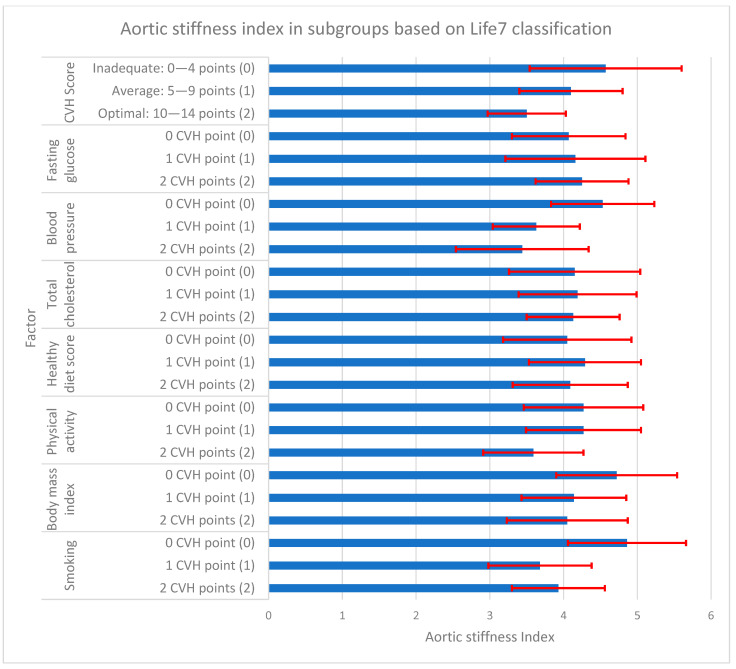
Aortic stiffness index in subgroups based on AHA Life’s Simple 7 classification. Vertical axis represents AHA Life’s Simple 7 cardiovascular health. The first part represents total CVH score, which is the sum of CVH points from the following 7 CVH factors. For each factor, participant obtains 0, 1, or 2 points and the total possible number is 14. Horizontal axis represents aortic stiffness index. The blue bars represent mean values of aortic stiffness index, whereas the red lines represent corresponding standard deviations. Numbers in () brackets (0), (1), (2) correspond to the same brackets in Table 4. CVH—cardiovascular health; AHA—American Heart Association.

**Figure 2:**
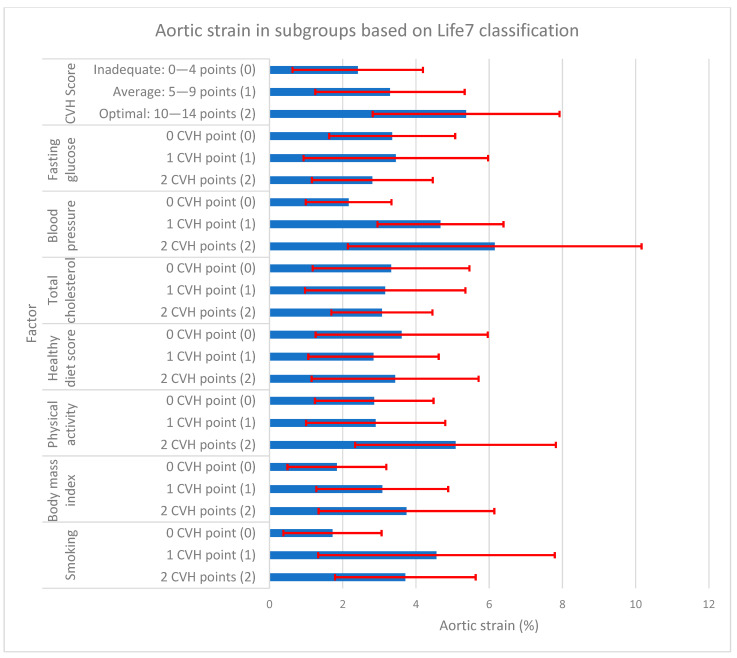
Aortic strain in subgroups based on AHA Life’s Simple 7 classification. Vertical axis represents AHA Life’s Simple 7 cardiovascular health. The first part represents total CVH score, which is the sum of CVH points from the following 7 CVH factors. For each factor, participant obtains 0, 1, or 2 points and the total possible number is 14. Horizontal axis represents aortic strain in %. The blue bars represent mean values of aortic strain (%), whereas the red lines represent correspond to standard deviations. Numbers in () brackets (0), (1), (2) correspond to the same brackets in Table 4. CVH—cardiovascular health; AHA—American Heart Association.

**Figure 3:**
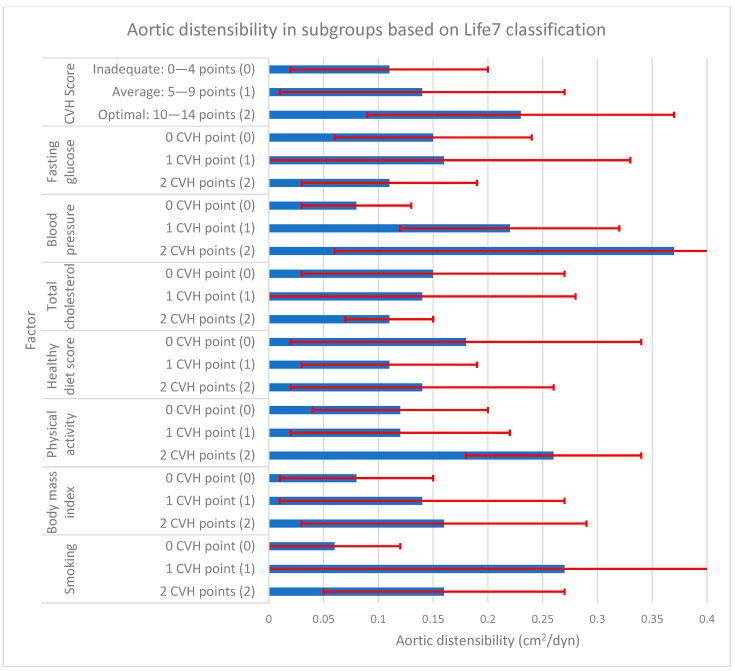
Aortic distensibility in subgroups based on AHA Life’s Simple 7 classification. Vertical axis represents AHA Life’s Simple 7 cardiovascular health. The first part represents total CVH score, which is the sum of CVH points from the following 7 CVH factors. For each factor, participant obtains 0, 1, or 2 points and the total possible number is 14. Horizontal axis represents aortic distensibility. The blue bars represent mean values of aortic distensibility (cm^2^/dyn), whereas the red lines represent corresponding standard deviations. Numbers in () brackets (0), (1), (2) correspond to the same brackets in Table 4. CVH—cardiovascular health; AHA—American Heart Association.

During our statistical analysis, we studied the relationship between aortic measurements (stiffness and elasticity) and CVH scores in subgroups differentiated by the number of obtained points for each ALS7 factor. The results of this analysis are presented in Figure 1, Figure 2 and Figure 3 and Table 4.

Based on the results, the aortic stiffness index (Ao stiffness index) was significantly higher in the group of current smokers, compared to participants who had quit smoking both less and more than 12 months before the study (4.86 ± 0.80 vs. 3.68 ± 0.70 and 3.93 ± 0.63, respectively). Conversely, it was noted that the aortic elasticity determined by the Ao strain and distensibility was lower in the actively smoking group in a similar comparison (1.72 ± 1.34% vs. 4.56 ± 3.23% and 3.71 ± 1.92% for Ao strain and 0.06 ± 0.06 cm^2^/dyn vs. 0.27 ± 0.30 cm^2^/dyn and 0.16 ± 0.11 cm^2^/dyn for Ao distensibility, respectively). Accordingly, smoking was associated with an increase in aortic stiffness and a decrease in aortic elasticity, and the results were considered statistically significant.

Analyzing the body mass index, the results were similar to those obtained in the group of smokers. However, they were not statistically significant in every subgroup. It was found that in the group with BMI ≥ 30 kg/m^2^, Ao stiffness was significantly higher than in the groups with a BMI of 25–29.9 kg/m^2^ and <25 kg/m^2^ (4.72 ± 0.82 vs. 4.14 ± 0.71 and 4.14 ± 0.71, respectively). However, in the case of Ao elasticity, statistically significant differences were obtained only in the comparison between the groups with BMI ≥ 30 kg/m^2^ and <25 kg/m^2^ (1.84 ± 1.35% vs. 3.74 ± 2.40% and 0.08 ± 0.07 cm^2^/dyn and 0.16 ± 0.13 cm^2^/dyn for Ao strain and distensibility, respectively). Therefore, a higher BMI was associated with higher Ao stiffness and inversely with lower Ao elasticity.

Unlike the two previously described measurements, it was found that patients presenting moderate-intensity physical activity for more than 150 min per week (or vigorous for more than 75 min per week or a combination of both) had significantly lower Ao stiffness than the groups with moderate activity 1–149 min per week (or vigorous and combination accordingly) and no physical activity at all (3.59 ± 0.68 vs. 4.27 ± 0.78 and 4.27 ± 0.81). On the contrary, it was found that aortic strain and distensibility were significantly higher in the group with the most minutes of activity per week in comparison to the two other groups (5.08 ± 2.74% vs. 2.90 ± 1.90% and 2.86 ± 1.62% for Ao strain and 0.26 ± 0.08 cm^2^/dyn vs. 0.12 ± 0.10 cm^2^/dyn and 0.12 ± 0.08 cm^2^/dyn for Ao distensibility, respectively). Differently from smoking and BMI, in the physical activity groups, higher activity was associated with lesser Ao stiffness and greater Ao elasticity.

No statistically significant associations were found between aortic parameters and healthy diet scores; similarly, no statistically significant associations were observed between aortic parameters and total cholesterol and fasting glucose levels.

It was found that the group with systolic blood pressure (SBP) of 140 mmHg or higher and/or diastolic blood pressure (DBP) of 90 mmHg or higher (0 CVH points) had a significantly higher aortic stiffness index than both groups with SBP 120–139 and/or DBP 80–89 or treated at that time to the goal (which was 1 CVH point) and of SBP/DBP < 120/80 mmHg (which was 2 CVH points), with the result being 4.53 ± 0.70 vs. 3.63 ± 0.59 and 3.44 ± 0.90, respectively. In the group with the highest SBP and/or DBP, significantly lower Ao strain and distensibility were observed than in both previously described blood pressure groups (2.16 ± 1.17% vs. 4.67 ± 1.72% and 6.15 ± 4.01% for Ao strain and 0.08 ± 0.05 cm^2^/dyn vs. 0.22 ± 0.10 cm^2^/dyn and 0.37 ± 0.31 cm^2^/dyn for Ao distensibility). In addition, it was found that the difference in distensibility between the group with the medium and lowest BP was also statistically significant (0.22 ± 0.10 cm^2^/dyn and 0.37 ± 0.31 cm^2^/dyn).

Analyzing the obtained total cardiovascular health scores (CVH score), there was a significantly higher Ao stiffness index in the group with inadequate CVH scores (0–4 points) in comparison to the average (5–9 points) and optimal ones (10–14 points), with aortic stiffness of 4.57 ± 1.03 vs. 4.10 ± 0.70 and 3.50 ± 0.53, respectively. For aortic elasticity, an inverse relationship was observed. There was significantly higher Ao strain in the group with optimal CVH scores compared to the average and inadequate ones (5.37 ± 2.55% vs. 3.29 ± 2.04% and 2.41 ± 1.78%). However, for aortic distensibility, a significant difference was noted only between optimal and inadequate CVH scores: 0.23 ± 0.14 cm^2^/dyn and 0.11 ± 0.09 cm^2^/dyn.

Correlations between aortic parameters and CVHS measurements were investigated. The results are presented in Table 5.

For the aortic stiffness index, there were significant positive correlations with the body mass index and systolic and diastolic blood pressure, and a negative correlation with the total cardiovascular health score. Subsequently, for aortic strain, there was a negative correlation with the body mass index and systolic and diastolic blood pressure, and a positive correlation with the CVH score. Furthermore, for aortic distensibility, a negative correlation was obtained with systolic and diastolic blood pressure and positive with the CVH score.

Estimation results for the model obtained in the multivariable stepwise backward regression analysis are presented in Table 6.

Interpretation of the regression models: in the ALS7 scale, higher scores in blood pressure, smoking, and BMI are independent protective factors against greater aortic stiffness. Higher scores in blood pressure, smoking, BMI, and physical activity are independent protective factors against lesser aortic strain. Higher scores of blood pressure and smoking are independent protective factors against lesser aortic distensibility.

## 4. Discussion

The results of the study showed statistically significant evidence supporting our hypothesis about the association between the decrease in aortic stiffness and an increase in elasticity in CCTA with an increase in cardiovascular health score measured by the ALS7. Higher aortic stiffness was found to be associated with smoking, a higher BMI, lower physical activity, higher blood pressure, and inadequate total cardiovascular health scores obtained in AL. In contrast, lower aortic elasticity was associated with smoking, a higher BMI, lower physical activity, and a lower CVH score.

Aortic strain is the deformation that results from the force applied to the given area of the vessel. Distensibility is the ratio between the deformation of the aorta under a given pressure level, and stiffness represents the resistance to the deformation [23].

The most reliable technique for the measurement of large arteries’ stiffness is the pulse wave velocity (PWV) and it is included in the guidelines of the European Society of Cardiology (ESC). Applanation tonometry is considered the gold standard. PWV measures the delay between the pulse time at the carotid artery and the femoral artery and accurately represents the arterial stiffness. PWV may help with the identification of groups of patients with higher cardiovascular risk. Its prognostic value for cardiovascular risk assessment has been documented by a wide variety of studies. For example, an increase of 1 m/s in PWV was associated with a 7% increase in the risk of a cardiovascular event for a 60-year-old male., On the other hand, the ESC guidelines note the practical difficulties of using PWV on a daily basis. Equally, it is emphasized in the Polish Hypertension Management Guidelines that only a few medical centers in Poland are capable of measuring PWV. Therefore, PWV may be particularly useful when there are extended indications, such as when screening hypertension-mediated organ damage [24,25,26,27,28,29].

The other means of measuring stiffness, which was used in this study, is computed tomography. No studies linking aortic stiffness and elasticity measured by CCTA to most cardiovascular health parameters can be found in the available sources. However, in one study, in the thoracic aorta, a decrease in aortic distensibility and strain and an increase in aortic stiffness were associated with higher age [30].

Among the reports to date, there are investigations focused on stiffness measured by PWV. Oyenuga AO et al. studied the relation between the ALS7 and stiffness measured by PWV. They associated lower arterial stiffness with greater AHA Life’s Simple 7 scores in the middle-aged population [31]. Similar results were obtained by Niiranen TJ et al., who studied the relationship between arterial stiffness measured by pulse wave velocity and cardiovascular health [32].

In this study, it was found that with an increase in blood pressure, aortic stiffness increases and aortic elasticity decreases. Although a study evaluating identical criteria could not be reached, it was noted in a cohort study on 30,384 patients that blood pressure was associated with stiffness measured by PWV, and higher systolic blood pressure trajectories over time were related to increases in stiffness [33]. Similarly, blood pressure was assessed in a group of patients with a transient ischemic attack or minor stroke. Elevated levels of SBP were associated with a greater rate of progression of pulse wave velocity [34]. Correspondingly, the relation between SBP and arterial stiffness was studied with similar results in a longitudinal study performed by Wu S. et al. on a group of 3277 participants during 2010–2016 [35].

The results of the present study associated higher aortic stiffness and lower elasticity with active and reverse outcomes for patients who quit smoking or did not smoke at all. Similar outcomes were obtained by Jatoi N. A. et al., who, in a group of 554 patients aged 18 to 80, assessed the relationship between smoking and arterial stiffness. They found that ex-smokers who quit smoking less than one year before the study had similar arterial stiffness to current smokers at that time. Those who quit smoking between 1 and 10 years before the study had intermediate levels of stiffness, and ex-smokers who quit more than 10 years before were not significantly different from non-smokers [36]. However, the present study observed a difference even in patients who quit smoking less than 12 months before the study. This may be caused by variations in the measuring technique, but further studies on this subject are necessary.

The statistical tests used in this study showed no significant difference between groups in terms of fasting glucose levels and aortic stiffness and elasticity. However, arterial stiffness was previously associated by Tian X. et al. with diabetes in a prospective cohort study [37]. Similarly, in Wildman R. P. et al.’s study, an association between glucose and arterial stiffness was observed [38]. This may have been caused by the underrepresentation of diabetic participants in the present investigation (only 11.4%).

Arterial stiffness has been associated with an increase in body weight in various studies [39]. In Wildman R.P. et al.’s study of 186 young and 177 older adults, a strong association between pulse wave velocity and BMI was observed [38]. In their study, groups were divided into those with a BMI of <25, 25–30, and >30, which was consistent with our study. For stiffness, we observed comparable results to the older participant group of Wildman et al.’s study. This was caused by the fact that our participants had a mean age 70.41 ± 8.32, which corresponded to Wildman et al., who studied a group with a median age of 60.5 in their older adult group. Similarly, Recio-Rodriquez studied the BMI’s association with arterial stiffness. However, it should be mentioned that he found that anthropometric parameters like waist circumference and the waist/height ratio were even better correlated with arterial stiffness than with BMI [40]. Similarly, abdominal obesity was associated with arterial stiffness in Fu S. et al.’s study in the Chinese population, and, as in the previously discussed study, the results of the waist circumference appeared to be more relevant than those for BMI [41].

The outcome of the study showed that actively exercising people had lower aortic stiffness and higher aortic elasticity. The relationship between physical activity and cardiovascular health parameters was previously studied in Park W. et al.’s research, in which, after 12 weeks of training, an improvement in blood pressure and arterial stiffness was observed in the group of obese men with BMI > 25 kg/m^2^ [42]. Similarly, physical activity was associated with lower arterial stiffness in other studies [43,44,45], although, in one study, an association was not observed among overweight and obese women [46]. Furthermore, the reducing effect of physical activity on arterial stiffness was confirmed by Lopes S. et al.’s meta-analysis, which included 14 trials [47], and this result is consistent with our study.

One of the objectives of the present study was to analyze the relationship between aortic parameters and the corresponding ALS7 cardiovascular risk factors. However, it should be emphasized that there are other important factors, such as non-HDL cholesterol, kidney function, and uric acid, which were not included in this study. In particular, non-HDL cholesterol is included in the Systemic Coronary Estimation 2 (SCORE2) algorithm and has a strong relationship with CV risk. Hypertension, dyslipidemia, and diabetes mellitus are prevalent among patients with chronic kidney disease, and chronic kidney disease (CKD) is an independent risk factor for cardiovascular diseases. Moreover, CV diseases are the leading cause of death among patients with CKD. Furthermore, as stated in the ESC guidelines, all patients with hypertension should be tested for uric acid levels routinely [48]. This study did not consider the impact of the above factors, as they were not included in the ALS7. Nevertheless, in the authors’ opinion, analyzing the influence of these parameters in further studies would undoubtedly be valuable. The strength of this study is in exploring the relationship between aorta measurements obtained from CCTA and cardiovascular health factors. However, the lack of identical studies to compare the results with limited the discussion and required us to compare our results with studies that did not use the particular measuring method that we used in this study. Although a sample size calculator was used and sufficient participants were recruited, the statistical tests failed to find significant differences in some parameters, like fasting glucose. Therefore, subsequent research on a larger group could be conducted to study some of these relationships further. Owing to the use of ionizing radiation, the methodology of the study carried limitations regarding the exposure of subjects to harmful agents. Therefore, this study was composed only of patients with medical indications to conduct CCTA, which means that the participants were a specific group compared to the healthy population. However, exposing healthy people to such an amount of ionizing radiation only to conduct a study would not be considered ethical.

In the absence of identical studies, the authors believe that the results obtained may be particularly useful due to their innovative nature and the fact that, to acquire the aortic measurements, an examination method that is already used in everyday practice can be adopted. Aortic stiffness and elasticity can by checked additionally to the usual CCTA and perhaps, after further studies, can be used as new clinical markers of CV health. The authors hope that the results of this study may prove useful in clinical practice. As mentioned above, although conducting PWV measurements is the gold standard in examining arterial stiffness, there are regions in which it is difficult to obtain. On the other hand, aorta measurements can be conducted during a routine CCTA. In view of the above, it is hoped that after further research, stiffness and elasticity assessments may be beneficial in clinical practice as part of cardiovascular health assessment.

## 5. Conclusions

Aortic stiffness and elasticity are associated with cardiovascular health parameters measured by the AHA Life’s Simple 7 questionnaire in a population composed of people with medical indications for coronary computed tomography angiography. Optimal scores of cardiovascular health are associated with decreased aortic stiffness and increased elasticity.

## Figures and Tables

**Table 1:** Clinical characteristics in the studied group.

	Whole Studied Group (*n* = 96)
Age (years)	70.41 ± 8.32
Height (cm)	166.90 ± 7.80
Body mass (kg)	71.52 ± 11.68
BMI (kg/m^2^)	25.58 ± 3.12
Gender (%)	
Men	54.2
Women	45.8
Smoking (%)	28.1
Arterial hypertension (%)	54.2
Systolic blood pressure (mmHg)	140.05 ± 18.56
Diastolic blood pressure (mmHg)	86.04 ± 9.15
Type 2 of diabetes (%)	11.4
Fasting glucose (mg/dL)	120.27 ± 49.31
Hypercholesterolemia (%)	67.7
Total cholesterol (mg/dL)	224.61 ± 43.25
Indication to CCTA (%)	
Chronic CAD suspicion	58.3
Chest pain	51.0
Numerous CAD risk factors	46.8
Low intermediate CAD risk	20.8
Inconclusive exercise test	15.6
Non-diagnostic exercise test	6.2
Regional wall motion abnormalities of left ventricular	3.1
Sudden cardiac death in the family history	1.0

BMI—body mass index; CAD—coronary artery disease; CCTA—coronary computed tomography angiography.

**Table 2:** Cardiovascular health parameters according to the AHA’s Life’s Simple 7 classification in the studied group.

AHA Life’s Simple 7 Classification		Whole Studied Group (*n* = 96)
Health behaviors		
Smoking (%)	Never or quit >12 months ago (2 CVH points)	65.6
	Former ≤12 months (1 CVH point)	6.2
	Yes (0 CVH point)	28.1
Body mass index (%)	<25 kg/m^2^ (2 CVH points)	39.6
	25–29.9 kg/m^2^ (1 CVH point)	48.9
	≥30 kg/m^2^ (0 CVH point)	11.4
Physical activity (%)	Moderate-intensity activity ≥150 min/week or vigorous-intensity activity ≥75 min/week or combination (2 CVH points)	14.6
	Moderate-intensity activity 1–149 min/week or vigorous-intensity activity 1–74 min/week or combination 1–149 min/week (1 CVH point)	50.0
	None (0 CVH point)	35.4
Healthy diet score (%)	4–5 components (2 CVH points)	11.4
	2–3 components (1 CVH point)	50.0
	0–1 component (0 CVH point)	38.5
Health factor		
Total cholesterol (%)	<200 mg/dL (2 CVH points)	11.4
	200–239 mg/dL or treated to goal (1 CVH point)	58.3
	≥240 mg/dL (0 CVH point)	30.2
Blood pressure (%)	<120/80 mmHg (2 CVH points)	5.2
	SBP: 120–139 mmHg and/or DBP: 80–89 mmHg or treated to goal (1 CVH point)	33.3
	SBP ≥ 140 mmHg and/or DBP ≥ 90 mmHg (0 CVH point)	61.4
Fasting glucose (%)	<100 mg/dL (2 CVH points)	35.4
	100–125 mg/dL or treated to goal (1 CVH point)	42.7
	≥126 mg/dL (0 CVH point)	21.9
Cardiovascular health score		
CVH score (point, mean ± SD)		6.56 ± 1.98
CVH score (%)	Optimal (10–14 points)	5.2
	Average (5–9 points)	72.9
	Inadequate (0–4 points)	21.9

AHA—American Health Association; CVH—cardiovascular health; DBP—diastolic blood pressure; SBP—systolic blood pressure; SD—standard deviation.

**Table 3:** Coronary computed tomography angiography parameters in the study group.

	Whole Studied Group (*n* = 96)
CACS	194.18 ± 59.48
CAD-RADS	
0	19.8
1	20.8
2	47.9
3	6.2
4	3.1
5	1.0
N	1.0
Ao diastolic diameter (mm)	33.33 ± 4.26
Ao systolic diameter (mm)	34.39 ± 4.39
Ao stiffness index	4.17 ± 0.80
Ao strain (%)	3.20 ± 2.08
Ao distensibility (cm^2^/dyn)	0.14 ± 0.13

Ao—aorta; CACS—coronary artery calcium score; CAD-RADS—coronary artery disease reporting and data system.

**Table 4:** Parameters of aortic stiffness and elasticity in subgroups based on the AHA Life’s Simple 7 classification.

AHA Life’s Simple 7 Classification Factor		Ao Stiffness Index	Ao Strain (%)	Ao Distensibility (cm^2^/dyn)
Smoking	2 CVH points (2)	3.93 ± 0.63	3.71 ± 1.92	0.16 ± 0.11
	1 CVH point (1)	3.68 ± 0.70	4.56 ± 3.23	0.27 ± 0.30
	0 CVH point (0)	4.86 ± 0.80	1.72 ± 1.34	0.06 ± 0.06
	*p* < 0.05	2, 1 vs. 0	2, 1 vs. 0	2, 1 vs. 0
Body mass index	2 CVH points (2)	4.05 ± 0.82	3.74 ± 2.40	0.16 ± 0.13
	1 CVH point (1)	4.14 ± 0.71	3.08 ± 1.80	0.14 ± 0.13
	0 CVH point (0)	4.72 ± 0.82	1.84 ± 1.35	0.08 ± 0.07
	*p* < 0.05	2, 1 vs. 0	2 vs. 0	2 vs. 0
Physical activity	2 CVH points (2)	3.59 ± 0.68	5.08 ± 2.74	0.26 ± 0.08
	1 CVH point (1)	4.27 ± 0.78	2.90 ± 1.90	0.12 ± 0.10
	0 CVH point (0)	4.27 ± 0.81	2.86 ± 1.62	0.12 ± 0.08
	*p* < 0.05	2 vs. 1, 0	2 vs. 1, 0	2 vs. 1, 0
Healthy diet score	2 CVH points (2)	4.09 ± 0.78	3.43 ± 2.28	0.14 ± 0.12
	1 CVH point (1)	4.29 ± 0.76	2.84 ± 1.78	0.11 ± 0.08
	0 CVH point (0)	4.05 ± 0.87	3.61 ± 2.35	0.18 ± 0.16
	*p* < 0.05	-	-	-
Total cholesterol	2 CVH points (2)	4.13 ± 0.63	3.07 ± 1.38	0.11 ± 0.04
	1 CVH point (1)	4.19 ± 0.80	3.16 ± 2.19	0.14 ± 0.14
	0 CVH point (0)	4.15 ± 0.89	3.32 ± 2.14	0.15 ± 0.12
	*p* < 0.05	-	-	-
Blood pressure	2 CVH points (2)	3.44 ± 0.90	6.15 ± 4.01	0.37 ± 0.31
	1 CVH point (1)	3.63 ± 0.59	4.67 ± 1.72	0.22 ± 0.10
	0 CVH point (0)	4.53 ± 0.70	2.16 ± 1.17	0.08 ± 0.05
	*p* < 0.05	2, 1 vs. 0	2, 1 vs. 0	2, 1 vs. 0; 2 vs. 1
Fasting glucose	2 CVH points (2)	4.25 ± 0.63	2.81 ± 1.65	0.11 ± 0.08
	1 CVH point (1)	4.16 ± 0.95	3.45 ± 2.52	0.16 ± 0.17
	0 CVH point (0)	4.07 ± 0.77	3.35 ± 1.72	0.15 ± 0.09
	*p* < 0.05	-	-	-
Total CVH score	Optimal: 10–14 points (2)	3.50 ± 0.53	5.37 ± 2.55	0.23 ± 0.14
	Average: 5–9 points (1)	4.10 ± 0.70	3.29 ± 2.04	0.14 ± 0.13
	Inadequate: 0–4 points (0)	4.57 ± 1.03	2.41 ± 1.78	0.11 ± 0.09
	*p* < 0.05	2, 1 vs. 0	2 vs. 1, 0	2 vs. 0

Table 4 presents mean values of aortic stiffness, strain, and distensibility with ± standard deviation among different cardiovascular health factors. In each factor, a subgroup of 0, 1, or 2 points is distinguished. The number in () parentheses is a subgroup number used for comparison. The CVH point criteria are defined in Table 2. Total CVH score represents the sum of points from all 7 categories above it. AHA—American Health Association; Ao—aorta; CVH—cardiovascular health.

**Table 5:** Correlations in the studied group.

	Ao Stiffness Index	Ao Strain (%)	Ao Distensibility (cm^2^/dyn)
Age (years)	ns	ns	ns
BMI (kg/m^2^)	0.20	−0.24	ns
Systolic blood pressure (mmHg)	0.61	−0.66	−0.69
Diastolic blood pressure (mmHg)	0.39	−0.49	−0.41
Fasting glucose (mg/dL)	ns	ns	ns
Total cholesterol (mg/dL)	ns	ns	ns
CVH score	−0.48	0.48	0.36

Table 5 presents Pearsons’s r correlation coefficients between each parameter of aorta and each measured factor. Only significant correlations are presented. Ao—aorta; BMI—body mass index; CVH—cardiovascular health; ns—non-significant.

**Table 6:** Results of estimation for the model obtained in multivariable stepwise backward regression analysis.

Model for: **Ao Stiffness Index**					
	Intercept	Blood Pressure		Smoking	Body Mass Index
Regression coefficient	5.211	−0.669		−0.409	−0.242
SEM of Rc	0.154	0.099		0.065	0.089
*p*	*p* < 0.001	*p* < 0.001		*p* < 0.001	*p* < 0.05
*p* of the model	*p* < 0.001				
Model for: **Ao strain** (%)					
	Intercept	Blood Pressure	Smoking	Body Mass Index	Physical Activity
Regression coefficient	0.526	2.019	0.807	0.461	0.339
SEM of Rc	0.041	0.245	0.158	0.219	0.096
*p*	*p* < 0.01	*p* < 0.001	*p* < 0.001	*p* < 0.05	*p* < 0.05
*p* of the model	*p* < 0.001				
Model for: **Ao distensibility** (cm^2^/dyn)					
	Intercept		Blood Pressure		Smoking
Regression coefficient	0.028		0.139		0.037
SEM of Rc	0.008		0.015		0.010
*p*	*p* < 0.01		*p* < 0.001		*p* < 0.001
*p* of the model	*p* < 0.001				

Ao—aorta; SEM of Rc—standard error of regression coefficient; blood pressure, smoking, body mass index, physical activity—nominal variables, where 2: 2 CVH points, 1: 1 CVH point, 0: no CVH point.

## Data Availability

The data that support the findings are available from the corresponding author upon reasonable request.
